# Building social accountability to improve reproductive, maternal, newborn and child health in Nigeria

**DOI:** 10.1186/s12939-022-01643-2

**Published:** 2022-04-07

**Authors:** Rachel Sullivan Robinson, Tariah Adams

**Affiliations:** 1 School of International Service & Accountability Research Center, American University, 4400 Massachusetts Avenue NW, Washington, DC 20016-8071 USA; 2 White Ribbon Alliance Nigeria, Abuja, Nigeria

**Keywords:** Social accountability, Maternal health, Reproductive health, Nigeria, Niger State, Health facility committees, Community participation, NGO

## Abstract

**Background:**

Like many places in Nigeria, Niger, a predominantly rural and poor state in the north of the country, has high fertility, low contraceptive prevalence, and high maternal mortality. This paper presents a descriptive, contextualized case study of a social accountability campaign run by the nongovernmental organization White Ribbon Alliance Nigeria to strategically mobilize collective action to demand quality maternal health care and improve government responsiveness to those demands. We treat maternal health as a component of reproductive health, while recognizing it as a less contested area.

**Methods:**

Data come from more than 40 interviews with relevant actors in Niger State in 2017 and 2018 during the initial phase of the campaign, and follow-up interviews with White Ribbon Alliance Nigeria staff in 2019 and 2021. Other data include White Ribbon Alliance Nigeria’s monthly reports. We analyzed these data both deductively and inductively using qualitative techniques.

**Results:**

During its first phase, the campaign used advocacy techniques to convince the previously reticent state government to engage with citizens, and worked to amplify citizen voice by hosting community dialogues and town halls, training a cadre of citizen journalists, and shoring up ward health development committees. Many of these efforts were unsustainable, however, so during the campaign’s second phase, White Ribbon Alliance Nigeria worked to solidify state commitment to durable accountability structures intended to survive beyond the campaign’s involvement. Key challenges have included a nontransparent state budget release process and the continued need for significant support from White Ribbon Alliance Nigeria.

**Conclusion:**

These findings reveal the significant time and resource inputs associated with implementing a strategic social accountability campaign, important compromises around the terminology used to describe “accountability,” and the constraints on government responsiveness posed by unrealistic budgeting procedures. The campaign’s contributions towards increased social accountability for maternal health should, however, also benefit accountability for reproductive health, as informed and empowered woman are better prepared to demand health services in any sector.

## Background

Nigeria has one of the highest rates of maternal mortality in the world—estimated in 2015 to be over 800 maternal deaths per 100,000 live births—and is the source of a fifth of maternal deaths globally [1]. High fertility and low contraceptive use contribute to maternal mortality rates, and are also driven by similar factors, such as lack of access to quality health care. In 2018, Nigeria’s total fertility rate was 5.3 children per woman and the modern contraceptive prevalence rate was 17% among currently married women [2]. In 2012, the Nigerian government made major commitments to decreasing these rates through the Saving One Million Lives initiative and the London Family Planning Summit, and over the past decade has received significant external resources for improving reproductive, maternal, newborn, and child health.

Most of these resources have gone to relatively conventional efforts to improve reproductive and maternal health that are common around the world, such as increasing the supply of contraceptives or the number of skilled birth attendants [3, 4]. But there are other strategies for improving the quality of health care that have the potential to improve reproductive and maternal health, including social accountability. Drawing from Joshi ([5]: p. 161), social accountability refers to non-electoral, citizen “efforts at ongoing meaningful collective engagement with public institutions for accountability in the provision of public goods.” Social accountability approaches ideally create space and means for citizens to hold providers and government accountable. Common techniques include advocacy for information and increased investment, community and citizen sensitization, budget tracking, and citizen score cards. Citizen score cards, for example, involve citizens and service providers working together to plan and prioritize the types of services provided, and then allow citizens to hold providers accountable by scoring them on how well they have provided those services [6]. Scholars have found mixed impacts of social accountability interventions on health outcomes, but many conclude that approaches that are strategic—increasing the capacity for both citizen action *and* government response to citizen demands—or integrated—cutting across multiple levels of actors or using multiple strategies—offer greater promise [5, 7–9].

Despite the growing body of evidence analyzing the impact of social accountability interventions on health outcomes, and particularly reproductive, maternal, newborn, child and adolescent health outcomes, scholars have identified gaps in knowledge. First and foremost, there is a need for more studies that analyze the social, political, and historical context in which social accountability interventions occur [10–12]. Second, given the complexity of the causal chain between a social accountability intervention and any health outcome, there is a real need to examine intermediary outcomes that relate particularly to the construction of accountability relationships [11]. Third, scholars have also pointed to a lack of research on strategic social accountability approaches [13], and a need to understand the nuances of implementation processes [14]. The descriptive, contextualized case study of a strategic social accountability intervention that follows begins to address each of these gaps. It describes the process through which a Nigerian nongovernmental organization (NGO) led an intervention to build social accountability and collective action around maternal health by both garnering government support as well as engaging citizens. The analysis takes into consideration the structural barriers and enablers the NGO faced in doing so, as well as how it adapted its campaign throughout the process in response to the social and political context and the demands of the involved citizens.

The case study below analyzes the campaign by the Nigerian NGO White Ribbon Alliance-Nigeria (WRA Nigeria) to foster social accountability for maternal, newborn, and child health in Niger State, Nigeria. WRA Nigeria’s campaign targeted both citizens and government, with the goal of ultimately improving health outcomes by increasing the quality, supply, and use of primary health care facilities. The analysis below covers two phases of the campaign. The first phase (years 1–3) focused on generating citizen demand and encouraging government response. The second phase (years 4–6), designed in response to the experiences of the first phase, shifted to working to establish more durable mechanisms for holding government accountable to its health care commitments.

Although the campaign did not directly address reproductive health insofar as it relates to provision of contraception and sexual health care services, since the 1994 International Conference on Population and Development, many in the global health community have treated maternal health as falling under the umbrella of reproductive health, understood to include the “right of access to appropriate health-care services that will enable women to go safely through pregnancy and childbirth and provide couples with the best chance of having a healthy infant” [15]. We take that approach in this paper.

Even without a big tent approach to reproductive health, however, the campaign’s focus on increasing accountability around primary health care services stands to ultimately benefit reproductive health care in the state because primary health care is a patient’s entry point to the health system and the main coordinator of care. Features of high quality maternal health care, such as the availability of drugs, respectful treatment of patients, and well-staffed clinics all motivate the use of primary health care facilities [16, 17], benefitting overall health, as well as reproductive and maternal health. Given that 74% of married women who use modern contraception in Niger State access it through the public sector [2], making public health facilities appealing is key to ensuring those who wish to use contraception will do so. Finally, as has happened globally and nationally in Nigeria, Niger State is beginning to explicitly group reproductive and maternal health, such as through efforts to develop a unified quality of care framework for reproductive, maternal, newborn, child and adolescent health [18]. That said, there is no doubt that sexual and reproductive health are more contested [19] than maternal health, with Nigeria as no exception [20], suggesting efforts to increase accountability for maternal health care may be less politicized. We return to this topic in the conclusion.

### Theory and literature on social accountability, health facility committees, and health outcomes

Following positive results in Uganda [21], the number of social accountability initiatives to improve health has increased. A recent systematic review of articles reporting on social accountability efforts in the health sector in African countries found the vast majority identified positive outcomes [22]. Community mobilization efforts, of which social accountability campaigns are an example, have also been associated with positive maternal and child health outcomes in sub-Saharan Africa [23]. In a study of a campaign in Gujarat, India similar to WRA Nigeria’s, researchers found increased rates of facility deliveries [24], and other research from India has shown community-based monitoring of maternal health services to be effective in building political capacity [25]. Schaaf et al. [26] found positive effects of World Vision’s Citizen Voice and Action approach—which combines community score cards with social audits—on health system responsiveness and the provision of health services in Zambia. Score cards were also associated with statistically significant increases in home visits during and after pregnancy, service satisfaction, and current use of modern contraceptives in Malawi [27].

Not all campaigns have, however, increased health care utilization or improved health outcomes [28]. Randomized controlled trials of a transparency and accountability program in Indonesia and Tanzania found no effect on the use of maternal and newborn health services, likely because citizen participation focused on activities with weak or indirect links to health outcomes [29]. Research also indicates significant barriers to pro-accountability efforts in many contexts, including limited health system capacity, insufficient funding for accountability programs, and inadequately localized understandings of accountability and the steps necessary for providers and managers to achieve it [22, 30].

Theoretical discussions around accountability present several key points relevant to the analysis that follows. First is the distinction between government response and responsiveness to citizen demands, which Fox describes as being on a continuum that leads to accountability [31]. “Response” refers to government promises, which may or may not be met, and is in contrast to “responsiveness,” which refers to government making good on its promises. Failures of responsiveness may be due to deceit in the initial response, but can also be the result of limited government capacity, or the absence of processes that increase citizen participation in decision-making [31]. Moving towards accountability thus requires the institutionalization of those processes, such that those in government cannot arbitrarily take them away or ignore them. As described in greater detail below, the first phase of WRA Nigeria’s campaign focused on responses, while in the second phase the NGO shifted its emphasis to building structures and processes intended to enable responsiveness and the move towards accountability.

Second, states must actually be able to respond to citizen demands for social accountability efforts to be effective [9]. For example, a functioning public financial management system is a prerequisite for successfully transitioning from response to responsiveness to responsive accountability [32]. According to Fölscher, “The [public financial management] system comprises the formal and informal structures, processes and rules by which resources are allocated to activities; by which needed goods and services for an activity are purchased, procured and paid for; and by which delivery of the goods and services (to result in the activity) are monitored” ([32]: p. 2). From this perspective, promises from public servants cannot possibly be anything more than responses in the absence of such a system. Budgets are at the heart of public financial management systems, and so budgets that are decoupled from actual funding disbursements make for a real roadblock in moving towards government responsiveness and accountability. Such is the case with Niger State’s health budget, as described in further detail below.

Third, social accountability efforts are fundamentally efforts to change power relations between citizens and the state [16, 33, 34]. This characterization is particularly true in the health field in settings where people receive health care in public clinics funded by the government, and power is especially relevant to accountability relations in the context of reproductive health [34]. Patient-provider interactions thus reflect and are impacted by broader relations of power that must be addressed when trying to increase accountability [33]. We return to this point in the conclusion when addressing the implications of social accountability for maternal health on accountability for reproductive health.

Alma-Ata enshrined the importance of community participation in the production of health and health care [35]. Generating, enhancing, and sustaining community participation is a key part of social accountability interventions, but can be challenging. The strength of civil society, extent of norms of collective responsibility, and the degree to which the community prioritizes the health issue at hand can all affect social accountability efforts [29, 36]. Citizens must also be willing and able to seek government accountability [37], which is challenging in non-democratic contexts or those with generally low levels of trust. Community participation in the social accountability intervention must also be meaningful, as opposed to tokenistic [22].

Health facility committees are frequently a focus of social accountability interventions in low- and middle-income countries, including sub-Saharan Africa [22], and played a role in WRA Nigeria’s campaign analyzed below. Health facility committees emerged out of broader efforts around decentralization and the Bamako Initiative with the goal to engage communities in health services decision making, in particular related to the use of funds generated from the sale of drugs [38]. Called different names in different places, these committees are supposed to enable social accountability by monitoring health facilities, as well as representing the interests of users in demanding accountability from health care providers. Scholars have found their ability to impact health outcomes to be mixed [38, 39]. Their effectiveness is often hampered by community members not knowing about their existence or purpose, being skeptical of their capacity to effect change, or feeling unrepresented by their membership [40]. Health facility committees often lack the capacity to sanction health care workers and are frequently composed of volunteers, and so even if engaged, may tend to focus on information dissemination and building collective action for self-help, rather than demanding accountability [41, 42].

Characteristics of the committees, of their communities, and of the facilities they manage influence their effectiveness [39, 43]. So too does the nature of their relationship with health workers, and the degree of support and capacity building government provides them [40]. Elements of the broader political system, and involvement by external NGOs in supporting health facility committees have also been found to impact their capacity [40]. Furthermore, committees must perceive themselves to be legitimate, and be perceived as legitimate by government, health workers, and the broader community in order to play any sort of accountability role [42].

In the analysis that follows, we heed calls to fill gaps identified by other authors who have studied accountability in the reproductive and maternal health fields. These include consideration of accountability processes at subnational levels, attention to the political context, the implementation process of accountability interventions, the degree and capacity of health care system responsiveness, the overall complexity of health systems, and the role of financial accountability [19, 24, 44].

### Context

**Fig. 1:**
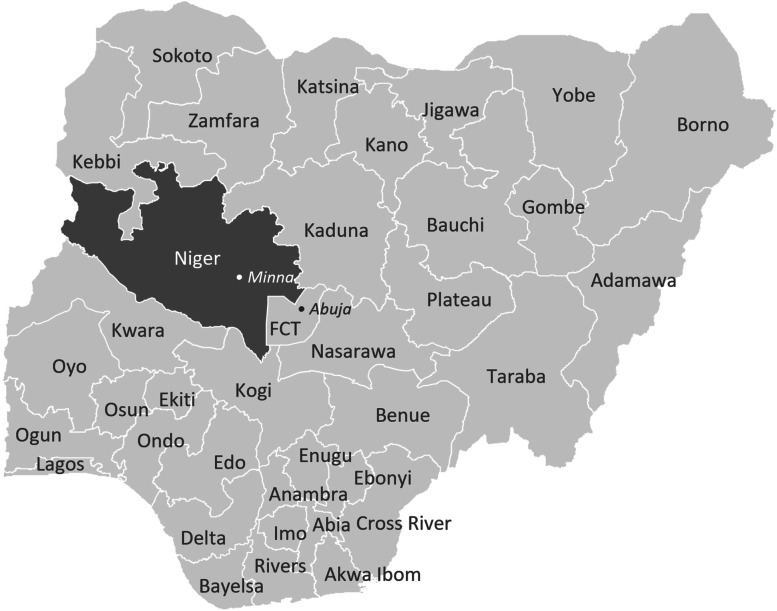
States of Nigeria. Source: Map produced by authors using KindofMap

**Fig. 2:**
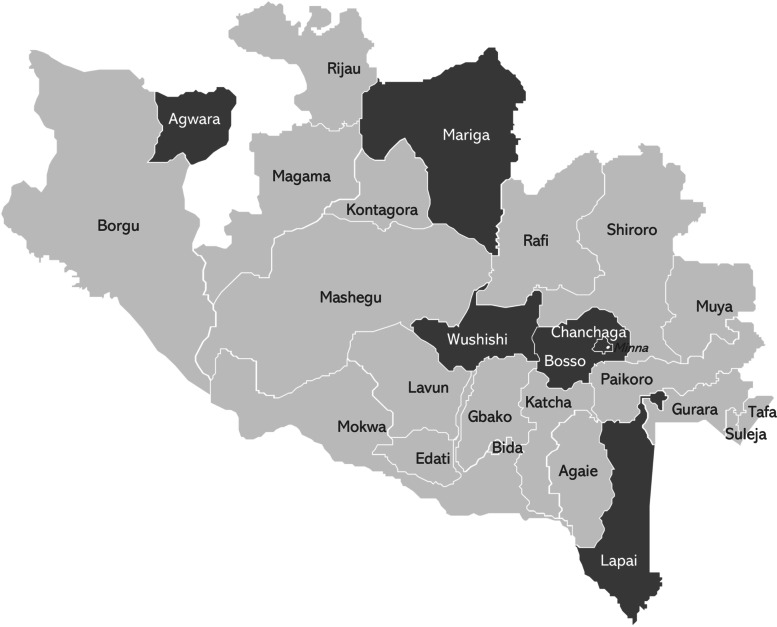
Local government areas of Niger State, Including White Ribbon Alliance target areas. Source: Map produced by authors using KindofMap

WRA Nigeria was founded in 2009 and is based in Nigeria’s capital, Abuja, with a field office three hours away in Minna, the capital of Niger State. A member of the Global White Ribbon Alliance, an international nonprofit organization, WRA Nigeria’s mission is “Activating a people-led movement for reproductive, maternal and newborn health and rights” [45]. The NGO has a staff of 12 (including interns) and an annual operating budget of approximately US$500,000. During the time period in question, the campaign in Niger State formed the bulk of WRA Nigeria’s activities, which also included national-level advocacy promoting reproductive and maternal health, citizen engagement in health, and transparency and accountability in the national health budget. Prior to the start of the social accountability campaign in Niger State, WRA Nigeria had no experience in the state. With funding from the Bill and Melinda Gates Foundation (the Gates Foundation) via the Global White Ribbon Alliance, in 2015 WRA Nigeria began a three-year campaign in three local government areas of Niger State—Chanchaga, Lapai, and Wushishi—to increase citizen-led accountability for maternal, newborn, and child health. WRA Nigeria received a three-year reinvestment from the Gates Foundation in 2018, which included expanding activities to an additional three local government areas: Agwara, Mariga, and Bosso (see Figs. 1 and 2 for more details). The campaign is multi-pronged, and specific activities have changed over time as WRA Nigeria has adapted to address challenges and updated strategic priorities. Broadly, the campaign seeks to build government support for improving maternal and primary health care quality, to enable citizens to monitor governmental follow-through on its responses (i.e., to move from response to responsiveness) and to undertake related advocacy, and to institutionalize accountability processes within governmental efforts to improve maternal and primary health care quality.

Located in Nigeria’s north-central geopolitical zone, Niger State’s population is 85% rural, approximately 80% Muslim, and Shari’a law has been practiced in parts of the state since 2009 [46]. The state has the largest land area among Nigerian states, and has many hard-to-reach areas. For example, WRA Nigeria’s target local government area of Agwara is a six-hour journey from Minna. There are three main ethnic groups—the Nupe, Gbagyi/Gbwari, and Hausa—and Hausa serves as a lingua franca. Almost half of the population of 5.3 million is under the age of 15, and approximately a third of the population lives below the poverty line [47].

Decades of military dictatorship as well as high levels of corruption have left Nigerians with low expectations for government, and although a democracy, voters do not hold full ability to sanction and reward elected officials [48, 49]. In addition, reliance on oil revenue, weak tax collection infrastructure, and a large informal economy mean the government collects, and citizens pay, few taxes [50], giving citizens in most states little stake in monitoring how the government uses revenue. Niger State is no different. Here, only 15 percent of the state government’s total revenue in 2018 was internally generated [51], which is not even enough to cover wages for state workers [52]. A 2015 survey demonstrated that citizens felt poorly engaged in governance and service delivery [53]. Fewer than five percent of respondents agreed that government informed citizens on how it spent money or that government regularly asked people what they thought of its plans to improve services. However, 73 percent agreed that they could express dissatisfaction with government services in public hearings where policymakers were present. Respondents also reported willingness to express their dissatisfaction with government to the press [53].

Poor maternal health in the state is the result of many factors related to both limited supply of high quality care and low demand for facility-based care, as well as high fertility (5.8 children per woman) and low contraceptive use (6.4%) (see Table 1).The main causes of maternal death in Nigeria are hemorrhage (23% of deaths), infection (17%), unsafe abortion (11%), toxia/eclampsia (11%), obstructed labor (11%), malaria (11%), and anemia (11%) [3]. Estimates from a 2013 survey of eastern Niger State suggest the same factors drive deaths there, and found that more than a third of women had experienced maternal mortality in their household [54]. A review of deliveries at the state’s tertiary and secondary hospitals indicated that delay in laboring women arriving at health care facilities is an important proximate cause of maternal mortality.^1^ The average travel time to a referral facility is 60–80 min [47], and people often must pay for services that should in principle be free [55]. As of 2018, more than forty percent of women in Niger State received no antenatal care, slightly less than a third of babies were delivered by a skilled provider, and approximately a quarter of children aged 12–23 months had received all basic vaccinations (see Table 1). Research from Niger State indicates a number of reasons why women choose not to deliver in facilities including a norm to deliver at home, the cost of transportation and care (despite maternal health services supposedly being free), poor treatment by staff, and cultural/religious barriers [54, 56].

**Table 1:** Indicators of women and children’s health and wellbeing in Niger State and Nigeria, 2018

	**Niger State**	**Nigeria**
*Women’s Status*		
Women literate (%)	25.9	53.1
Women who agree that wife-beating is justified (%)	62.4	28.0
Women in polygamous marriage (%)	40.4	30.5
Median age at first marriage (among those 20–49)	17.7	19.1
Women with no weekly exposure to media (%)	68.6	55.6
Married women mainly/jointly make decisions about family planning	68.3	89.5
*Fertility and Contraceptive Use*		
Total fertility rate	5.8	5.3
Median age at first birth (among women 25–49)	19.3	20.4
Women 15–19 who have begun childbearing (%)	26.1	18.7
Contraceptive prevalence (modern, %)	6.4	12.0
Unmet need for contraception (%)	19.2	18.9
*Maternal Health*		
HIV prevalence 2019 (%)	0.7	1.4
No antenatal care (%)	40.7	24.4
Took iron during last pregnancy (%)	60.3	69.3
Protected against neonatal tetanus (%)	37.2	52.9
Received intermittent malarial protection during pregnancy (%)	44.2	63.6
Facility deliveries (%)	25.8	39.4
Delivered by skilled provider (%)	30.6	43.4
Households with at least one insecticide-treated net (%)	46.9	60.6
*Infant Health*		
No postnatal checkup (%)	76.2	60.4
Children 12–23 month having received all basic vaccinations (%)	23.3	31.3
*Economic Development*		
Houses with improved drinking water source	61.0	65.3
Households with improved, not shared sanitation facility	38.5	53.4
Households with fixed or mobile handwashing station	57.7	81.1
Soap available	11.0	37.5

2018 Demographic and Health Survey [2], National Agency for the Control of AIDS [57]

Of those delivering within facilities, 93% do so in public facilities

Government commitment to primary health care has increased in Nigeria over the past decade. The 2014 National Health Act led to the creation of new agencies and funding streams. Niger State is ahead of most other states in reorganizing all primary health care functions under the State Primary Health Care Development Agency, which oversees implementation at the local government and ward level. In fact, by 2018, Niger State had the second-highest rating among all states in the country for Primary Health Care Under One Roof metrics [58]. The state launched a health plan, Niger Health 1.0, in 2016 [59], that outlines how to implement the National Health Act and includes a commitment to a functioning primary health care facility in each ward of the state.

As a result of this plan, Niger State was selected along with only two other states (Abia and Osun) to pilot the Basic Health Care Provision Fund element of the National Health Act, with support from the World Bank and the Gates Foundation.^2^ The National Health Act calls for one percent of Nigeria’s general revenue to be available for primary health care, or approximately N35 billion per year (US$115 million) as of 2016 [60]. Half of the fund is intended to provide a package of basic primary health care services through the National Health Insurance Scheme, another 45% to the National Primary Health Care Development Agency for the purchase of drugs, and the remaining 5% for emergency response [60]. The particular focus on health insurance is designed to reduce out-of-pocket health expenditures, which in Nigeria are among the highest of any country in Africa [61]. To receive money from the Fund, state and local governments must match at 25% [60]. Although the National Health Act was passed in 2014, this funding element was included in the federal budget for the first time only in June 2018 [62], funds were not released until late in 2019, and facilities in Niger State did not receive them until the second half of 2020.^3^ Deep concerns exist about the management of the Fund, with steep challenges to accountability related to the broader landscape of corruption in Nigeria, but also the actual mechanics of transferring money from the federal to the ward level [60]. Concerns also exist about Niger State’s monitoring capacity more broadly, as the state’s primary health care facility performance monitoring system was described in 2016 as “weak, irregular and uni-directional” ([53]: p. 39).

Tracking the health budget in Niger State is difficult. The percentage of the state’s budget allocated to health has increased steadily in recent years, from eight percent in 2015 to closer to 12 percent in 2017 [47]. This figure is still below the 15 percent suggested for national budgets by the Abuja Declaration, but more relevantly, does not necessarily translate into actual funding for health as the state ultimately releases quite a low percentage of budgeted amounts [58], in recent years less than 20 percent.^4^ Although maternal, newborn, and child health has no line item in the state budget, primary health care does—almost 30% of the total in 2018—but staff must apply for these funds with a memo, and the funds are released only in tranches [58]. In part because of these shortages, but also perhaps facilitating them, the state depends heavily on NGOs and donors to implement health programs [58, 63]. Similar financing difficulties will also likely hamper the state’s effort to provide the health insurance component of the Basic Health Care Provision Fund [61].

Nigeria’s primary health care provision system is decentralized. Niger State has 25 local government areas (see Fig. 2), which are second-level administrative units roughly equivalent to US counties. Each local government area is divided into approximately 10 wards, with a total of 274 wards in the state. The state has about 1,100 health facilities, including centers, clinics, and posts.^5^ The quality of primary health care facilities in Niger state is lower than average compared to other Nigerian states [64], but varies greatly among locations, with rural and hard-to-reach areas the most likely to have limited drugs and equipment. Each ward is supposed to have a fully functional facility (doctor, nurse, lab equipment, electricity, etc.), but as of 2017, only one met the full “functioning” criteria [47]. A 2017 primary health care assessment survey also found that approximately a quarter of staff were absent from post [47]. WRA Nigeria’s 2019 assessment of health care facilities located in nine local government areas found that no more than two-thirds met any one of the seven quality, equality, and dignity standards [58]. In particular, only 13% of facilities had adequate/reliable power supply, only 25% had water, sanitation, and hygiene (WASH) facilities, and about 40% of essential drugs had been stocked-out at some point in the six months prior to data collection [58]. Other issues faced by facilities included inadequate means for transferring patients to higher levels of care, lack of space for confidential consultations, and insufficient female staff [58]. Corroborating these statistics, when WRA Nigeria asked midwives from the state in late 2020 what they most wanted, the requests included equipment, supplies, better salaries, and more staff.^6^

Prior to WRA Nigeria’s campaign, there was little citizen involvement in health-related decision making in Niger State. Although most wards had ward development committees—mandated since 2000 to institutionalize community participation, particularly in health—these committees served as a political mechanism to receive and dispense patronage, not as sites of citizen action. Broader health care reforms in Nigeria led to the expectation of the creation of ward health development committees, health facility committees at the ward level intended to articulate community health needs, identify resources to meet them in conjunction with government and NGOs, and supervise the health facility, including use of any drug revolving funds [42]. Notably, national guidelines do not explicitly give these committees responsibility for accountability [42]. Analysis of ward health development committees in four Nigerian states found that they suffered from many of the same challenges faced by health facility committees described in the literature review above, and that only approximately half operated as government “botherers,” with the rest functioning as community conveners and substitutes for government [42]. The release of the Basic Health Care Provision Fund has amplified their role, however, as committee members are signatories to the account that receives funds each month, and communities are supposed to decide how the funds are spent.

Given the importance of social, political, and historical context to the implementation of social accountability interventions, several points about Niger State are worth highlighting. First, the state government is not particularly accountable to its citizens, and citizens have a long (and justified) history of mistrust of government. Second, the capacity of government facilities to provide health care is limited given the insufficient budget release. Third, there are some factors countering these first two trends, namely the existence of the ward health development committees, and an increased commitment to public health provision in Niger State mirroring broader national trends.

## Methods

The data for this analysis come from 42 interviews conducted by the authors in 2017 and 2018 in Minna, Niger State, as well as an additional three follow-up interviews conducted by the first author with staff from WRA Nigeria and the Global White Ribbon Alliance Secretariat in 2019 and 2021. The Minna interviews were with citizens (3), citizen journalists (7), religious and traditional leaders (3), civil society organizations and NGOs (7), the Niger State Ministry of Health (14), health care providers (2), elected officials (2), and WRA Nigeria staff (4). Two of the three interviews conducted with citizens included several people, as did two of the civil society interviews. Ten of the Minna interviews were with the same person, providing a valuable longitudinal perspective. Each interview lasted approximately 45–60 min and questions centered around respondents’ understandings of the drivers of poor maternal, newborn, and child health in the state, their interpretation of citizen-led accountability, how government had responded to citizen-led accountability, the barriers to increasing accountability, and their impressions of the WRA Nigeria campaign. All but two were conducted in English; the other two were conducted entirely or partially in Hausa, with translation provided by someone else present during the interview. The study was approved by American University’s Institutional Review Board and all respondents gave consent to participate as well as to be quoted. Information from interviews is referred to with a number and a note indicating the type of respondent.

Additional data came from articles published in print and online sources in Niger State, as well as WRA Nigeria monthly reports, which are for internal monitoring and evaluation purposes. These reports describe key activities and events, and provide process-related details, such as the number and type of stakeholders attending events. They also provide qualitative description of these activities, and of the broader context, such as related governmental activities to improve maternal health care quality in Niger State. We analyzed these data with NVivo through an iterative process, coding first for pre-determined themes (e.g., conflict between WRA Nigeria and the government and its resolution) and then developing new themes inductively.

The positionality of the authors is important to address before turning to the analysis, given that the first author was contracted by the White Ribbon Alliance Global Secretariat to conduct the case study and the second author, who facilitated the selection of interview respondents, was a WRA Nigeria employee. Such “closeness” to the subject matter appropriately raises concerns about bias, although we do not believe the analysis below to be unduly biased for several reasons. First and foremost, the analysis is a descriptive, contextualized case study, not an impact evaluation, so we have worked to focus on the details of implementation made possible by that insider perspective. The first author never felt any pressure from the Global Secretariat to produce positive results. Second, the case study details that WRA Nigeria faced significant barriers in implementing their campaign, not all of which they could overcome, suggesting that respondents did not provide the authors with a uniformly positive perspective. Third, there is quite a bit of published research about NGOs written by people who work for them or funded by them, both in the social accountability field [26, 65], but also more broadly [66], so we do not deviate from established practice.

## Results

We present results according to three key areas of WRA Nigeria’s strategic social accountability campaign: building government support, enabling citizens, and institutionalizing accountability processes. We also briefly discuss some of the outcomes, based on anecdotal evidence, attributed to the campaign.

### Building government support

By the end of the first phase of WRA Nigeria’s campaign, a number of key individuals had made statements and/or taken actions that demonstrate commitment to some form of citizen involvement in the health care provision process. Key individuals include the Commissioner of Health (the head of the State Ministry of Health), the executive director of the State Primary Health Care Development Agency, the First Lady of Niger State, and leaders in the state assembly. Traditional leaders have also played a role in encouraging the bureaucratic support for citizen involvement. That said, many of these commitments qualify as responses in Fox’s framework [31], as opposed to responsiveness or full accountability.

The state government was initially skeptical of WRA Nigeria’s campaign. In particular, they were used to working with service provision NGOs, with whom they would set up a memorandum of understanding and then leave alone. WRA Nigeria does not, however, provide services, so the state had no prior model to structure its relationship with them. Because of the focus of WRA Nigeria’s campaign on accountability, state actors also had concerns that WRA Nigeria might be anti-government, or in support of the opposition party. Thus WRA Nigeria spent the initial year of the first phase of the campaign almost exclusively building trust and securing buy-in among key state actors, which staff accomplished through repeated advocacy visits, patient explanations of the campaign’s goals, and leveraging early supporters to win over those who were more skeptical. In particular, WRA Nigeria focused on the State Ministry of Health and the Primary Health Care Development Agency, given the key roles these entities play in quality of care and the administration of the Basic Health Care Provision Fund, respectively.

Most people involved with primary health care see a role and need for community involvement in health, which created a helpful point of entry for the campaign. In particular, the key agency within the Ministry of Health tasked with primary health care, the Primary Health Care Development Agency, has been particularly supportive of the campaign. Its executive director at the start of the campaign, Dr. Yahaya Nauzo, was a doctor originally from Niger State who had practiced medicine in the US for many years and became an early backer of the campaign. Other important support came from the First Lady of Niger State, Dr. Amina Abubakar Bello, an OB/GYN who still sees patients at the general hospital and has an NGO, the Raise Foundation, which supports maternal health. She has provided support to the campaign throughout its existence, and has made public statements in support of accountability. Traditional and religious leaders facilitated WRA Nigeria’s early interactions with the Commissioner of Health. These included the Emir of Minna, the most important religious leader in the state and who has an overarching commitment to health, and a traditional leader, Alhaji Abdullahi Galadima Kagara, who honed an interest in health during participation in earlier polio vaccination campaigns.

WRA Nigeria’s staff members expended significant time and energy to cultivate the support of high-level individuals in the state. The two staff members most frequently on the ground in Niger State, the program manager and communications officer, both speak Hausa and spent countless hours in dialogue. Respondents affirmed this need for persistent and continuous interaction with high-level individuals, but noted interactions should not always focus explicitly on campaign goals. Respondents felt it more important to first build relationships with these individuals, so that advocacy will be taken seriously. As an elected official put it, “Most times, government doesn’t respond immediately to a campaign. They need to see first if the program is serious. Persistence is key–you need to keep coming back. If you don’t come back, it seems your program is not serious.”^7^ A traditional leader described persuading government officials as follows: “You need to make friends, not girlfriends, with those in power. Come say hello, but stay no more than 15 min. Know the club that they attend, and go discuss with them there. They may listen more at the club than elsewhere.”^8^

Early in their relationship building, WRA Nigeria realized that they had to educate policymakers about accountability, the same as other citizens. In particular, some officials were concerned that they did not have the authority or capacity to respond to people’s demands. To counter this concern, WRA Nigeria staff argued that officials could use knowledge of citizens’ needs to lobby the state and health bureaucracies for additional resources.

WRA Nigeria also tried to support health budget release. As described in the context section, budget release enables government’s ability to be responsive. A respondent noted that the state tended to release funds when donors offered matching funding that required the government to release its own funds first.^9^ Budget release also depends on program officers within the State Ministry of Health who must write proposals for funds to be released. Those who write better proposals tend to get more funds, so WRA Nigeria has provided support with proposal writing. A respondent working for a health care financing partner NGO noted that in order to ensure that more money is released, agencies need to both (a) have a plan so that they can quickly ask for money when it becomes available, and (b) spend the money they are given so they are justified in asking for more.^10^

Bringing the government on board required WRA reframing “accountability” to government officials as something of benefit to government. WRA Nigeria’s campaign goal was initially phrased as increasing “citizen-led accountability” for maternal, newborn, and child health services, but they purposefully switched to using “citizen engagement” in Niger State as a compromise to facilitate working with the government, whose own term is “community action for health.” To many in Niger State, “accountability” sounds like a financial term. As a WRA Nigeria staff member explained, “‘accountability’ sounds aggressive, offensive; ‘engagement’ is better. WRA Nigeria tries to be subtle in its messaging. ‘Accountability’ is a bitter pill to swallow–always thinking you’re talking about how much money, what it’s spent on, and so forth.”^11^ Reflecting this interpretation, a traditional leader explained, “People hear ‘accountability’ and they think money, corruption (especially), and everyone wants to run–you won’t get cooperation. You need to explain what is meant. Talking about citizens and government working together is the best thing to do.”^12^

Differences in interpretation of the meaning of “accountability” between WRA Nigeria and the government hampered the first year of WRA Nigeria’s campaign. An early campaign brief reported,

The concept of citizen-led accountability is quite foreign to many people, including policy makers, and most of them found the concept unfriendly and were concerned it would instigate the masses to turn against them. We had to explain the benefits of citizen-led accountability to them and assure them we were working toward a mutually-beneficial relationship for citizens and the government. [67]

In order to address negative interpretations of accountability, WRA Nigeria brought people together to identify barriers. They found that the media also primarily understood accountability in terms of budgets for health and the associated release of funding. As a result, the communications officer explained citizen-led accountability as being about finding solutions, and emphasized that citizen opinion and feedback are a critical resource for policy formulation and implementation. She also held a meeting with policymakers to say that accountability is not *only* about budgeting, but that maternal, newborn, and child health problems are better served when everyone comes together and talks. WRA Nigeria has also stressed their willingness to cooperate with the state’s strategic plans in the health sector, and has noted that because greater accountability should ultimately improve health care quality, it will also help increase health care utilization, contributing to achieving the state’s health care utilization strategic goals.

Within its own documents, the Ministry of Health uses the term “community action for health” which refers to community ownership of, and involvement with, health care. The government has come to understand this term as synonymous with WRA Nigeria’s “citizen engagement.” While WRA Nigeria ultimately accepted the substitution of “community action for health” for “citizen engagement,” they worried that the government would revert to solely gathering information, as opposed to participating in platforms to identify joint solutions for development and action planning with real potential for holding leaders accountable.

### Engaging citizens

Citizens in Niger State have indicated their willingness to hold government to account for promises made regarding health care, but have had limited opportunities to do so. Plenty of frustration exists with the quality of maternal, child and newborn health care in Niger State. Many respondents described incidents of poor care that either they or family or close friends had experienced. As one citizen journalist explained, “It doesn’t take much convincing for people to demand rights–it’s not as though they don’t know about what they’re experiencing. Some say they can’t talk back at the government, but I say you can.”^13^ Similarly, a traditional leader reported, “People are willing to complain, but they don’t know how to start because they don’t know their rights.”^14^

WRA Nigeria developed three mechanisms in the initial phase of their campaign for citizens to voice demands and perspectives: community dialogues, town halls, and citizen journalists. Community dialogues were primarily for the community alone (although sometimes had lower-level members of government present) while town halls brought together citizens and representatives of government. WRA Nigeria targeted leaders from within civil society as well as organized citizen groups to attend both types of meetings. These included youth leaders, traditional and religious leaders, women’s groups, farmers’ associations, and teachers’ associations. Their intent was to ensure that individuals and organizations representing the various constituencies within communities could carry forward the needs and demands of citizens, as well as signal to decision makers via their social and political power that those demands should be taken seriously. The town halls and community dialogues were very popular among citizens as well as individuals lower in the state government hierarchy and in parallel authority structures (religious and traditional leaders) because it gave them better access to those with power. Within the first three years of the campaign, WRA Nigeria hosted seven community dialogues (with an average of 375 people each) and five town halls (with an average of 550 people each) across the initial three focal local government areas.

Community dialogues were designed for community leaders and citizens to meet and identify the barriers to health access in the community and brought together the groups listed above as well as health providers. At these meetings the community collectively came up with their health demands and possible solutions. At town hall meetings, decision makers from the Sate Ministry of Health and Primary Health Care Development Agency listened to citizen demands and then co-designed a feasible action plan to meet those demands. Copies of the action plan were given to the community leaders, WRA Nigeria, and the State Ministry of Health and the State Primary Health Care Development Agency. The action plan fed into the state operational plan for health, and guided WRA Nigeria’s advocacy activities.

Community dialogues and town halls were largely facilitated by WRA Nigeria, and sustaining them proved to be difficult, as the refreshments and reimbursement for transportation crucial to ensuring participation were non-negligible costs, and WRA Nigeria’s plan for the government to take over hosting them did not materialize. As someone in the Ministry of Health stated bluntly, “Government is struggling to put workers in facilities–it’s too much to ask government to do town halls in every community as well.”^15^

Citizen journalists were another strategy to amplify citizen voice given overwhelming state ownership of the media in Niger State and low prioritization of health reporting. During the first phase of the campaign, WRA Nigeria trained approximately 30 people, four of whom were professional journalists, on maternal health issues, how to write human interest stories, using pictures to tell stories, partnering with the media, how to plan community meetings, how to access policymakers, and ethics in journalism. WRA Nigeria created this program out of a belief that poor reporting on maternal, newborn, and child health issues limited decision makers’ responsiveness to demands to improve health services, to help monitor health services as well as celebrate examples of government responsiveness, and in order to generate real-life stories to use for advocacy purposes. WRA Nigeria selected and trained these journalists; in some cases the health educator from the local primary health care center assisted with identifying suitable candidates. Journalists had to have at least some higher education, and although WRA Nigeria intended to have equal gender representation, it proved difficult to engage and retain women given competing priorities for their time and cultural expectations about appropriate activities. Of the 20 citizen journalists who remained after two rounds of training, 14 were men and six were women.

After a citizen journalist wrote a story, WRA Nigeria staff then edited the stories and helped publish them, primarily in online forums. Citizen journalists did not receive any compensation for their work, but WRA Nigeria occasionally funded their travel to hard-to-reach areas of the state. Citizen journalists’ most important contribution was tracking the state health budget, carried out by the professional journalists. This tracking required struggling to obtain copies of the detailed state budget.^16^

A small cadre of particularly enthusiastic citizen journalists organically emerged who WRA Nigeria then helped foster further and who they call “super-mobilizers,” individuals active in their communities, desiring change, and interested in writing as well. Super-mobilizers “can get people [to come out]. Being a citizen journalist brings them respect, relevance, and trust. People tell [the citizen journalists] their health problems.”^17^ One super-mobilizer carried out a number of watch-dog activities, including visiting the homes of families with newborns in his neighborhood and asking for details about the delivery.^18^ Although the citizen journalists were not intended to be key players in community meetings and town halls, super mobilizers contributed to these fora because they had in-depth knowledge of health care experiences in their communities and were motivated to speak up. For example, one citizen journalist persisted in speaking at a town hall even after a senior government official tried to silence him.

Citizen journalists reported being personally impacted by engagement with WRA Nigeria. One stated, “I know my rights as a human being because of work with White Ribbon.”^19^ Another noted that being a citizen journalist had made him the go-to source when community members found new problems with health facilities.^20^ Another citizen journalist also reported trickle-down effects of the training she had received from WRA Nigeria. “When we are mobilizing, I tell people to go for antenatal [care], to go the hospital for delivery. We talk to the leader of the village before mobilizing. We do community dialogue the way White Ribbon taught us. Now we teach them, and they are responding. I tell my own story.”^21^

The citizen journalist concept was innovative, but challenging to implement. New citizen journalists needed to be trained after a number moved away from the area during the first year of the effort. WRA Nigeria staff spent a great deal of time editing the stories of the non-professional citizen journalists to help them be suitable for publication. Although citizen journalists indicated that community members informed them of health issues, residents were not always willing to share stories about negative health experiences given that doing so often meant reliving traumatic events. Journalists also varied in their willingness to call out the government; as one explained, “Some citizen journalists don’t want to write reports because of the implications for them as they are working for the government indirectly in some way.”^22^

Given these challenges, during the second phase of the campaign, WRA Nigeria thus shifted away from citizen journalists to community volunteers, some of whom had been citizen journalists. Community volunteers work within the community to capture women’s experience of care, track the performance of several ward health development committees, and monitor the implementation of the Basic Health Care Provision Fund. Community volunteers also work with mainstream journalists to identify stories around health care demands or improvement within the community, and data from questionnaires they administer provide WRA Nigeria with a better understanding of current realities within the communities. For now, WRA Nigeria pays the community volunteers a stipend, but the long-term plan is to integrate them into state programs focused on volunteers. The volunteers have a monthly meeting to review their work, discuss challenges, and identify solutions, and also have a WhatsApp group so that they can easily troubleshoot with one another.

WRA Nigeria worked with local government area health educators to identify community volunteers: each must have a secondary school certificate, be respected in the community, have experience with community mobilization, be passionate about maternal health, and have a good relationship with health facility staff. Maintaining that good relationship can be hard, as health workers are often suspicious of the volunteers given that they report directly to WRA Nigeria. WRA Nigeria sought to identify a mix of men and women, but found it difficult to identify women that fit the criteria, so the volunteers are primarily men. The WRA Nigeria communications officer calls each one every week in order to help keep them motivated.

Another new way in which WRA Nigeria engaged citizens during the second phase of their campaign was through implementation of the Global Secretariat’s What Women Want Campaign. The purpose of this global campaign is to solicit the one thing women want most for their own reproductive and maternal health care; WRA Nigeria helped with the implementation of the campaign in Nigeria, including in Niger State and with the use of citizen journalists there [68]. The campaign was nation-wide and entailed surveying over 78,000 women and girls accessing healthcare about their top priority for maternal health care. Staff were surprised to learn that that the main thing women wanted in Niger State was WASH at primary health care centers, in particular better toilet facilities [68]. This demand had not emerged from any of the previous community dialogues, although community dialogues had identified respectful maternity care as a priority, as did the What Women Want campaign [68]. As a result of these findings, WRA Nigeria is now doing advocacy around WASH, in addition to other priorities, which has turned out to be much easier than promoting accountability. As one WRA Nigeria staff member explained, “Accountability was a struggle. It was antagonizing, and people would say, ‘You don’t understand our problems.’ Now [with WASH] everyone is on the same page. Stakeholders were engaged from the beginning because it’s about their people, coming from their people.”^23^

### Institutionalizing accountability processes

At the end of the first phase of the campaign, WRA Nigeria realized that much of their effort had gone towards organizing and facilitating community dialogues and town halls. Although the government claimed they would respond to demands made through these vehicles, little had happened: citizens came up with plans, but they needed to be funded, and the state never had the money. The government thus gave a response, but was not responsive [31]. So WRA Nigeria decided instead to focus their efforts on trying to strengthen the broader accountability system so that it could function, regardless of WRA Nigeria’s presence.

During the second phase of the campaign, WRA Nigeria focused on institutionalizing accountability mechanisms for the disbursement of the Basic Health Care Provision Fund. First, in collaboration with other NGOs, WRA successfully advocated for the elaboration and adoption of a Niger State Basic Health Care Provision Fund Accountability Framework. The Accountability Framework outlines the roles and responsibilities of key stakeholders, including governmental and community-based actors. WRA Nigeria provided technical support and advocacy for the creation and inclusion of extensive community participation mechanisms, including community listening sessions led by ward health development committees, and a score card that a coalition of civil society organizations will complete twice a year. Moreover, to maximize accessibility and transparency of information regarding the implementation of the Basic Health Care Provision Fund, the Primary Health Care Development Agency and WRA Nigeria are working with a consultant to create a digital dashboard to complement the paper-based system. Ultimately, ward health development committee members should be able to log in to the digital tool and report on Basic Health Care Provision Fund implementation. The digital dashboard is expected to be launched in October 2022. In the meantime, data are being collected on paper at the facility and ward level and aggregated at the local government area and state level. Community input is reflected through a “Community Engagement and Redress Tracker.” Among other things, this tracks community outreach by the primary health care center; whether or not the center holds meetings in which communities participate; and feedback from the community (gathered by the ward health development committee) on whether they were charged for services, if they were treated with dignity, and if staff were courteous and respectful.

WRA Nigeria has also worked to institutionalize accountability processes through their support for ward health development committees. As part of the first phase of the campaign, WRA Nigeria along with UNICEF helped to “reactivate” many of the state’s ward health development committees. Reactivation included setting up committees where none existed, ensuring that committees were formed according to the national guidelines (e.g., at least 40 percent women, not all members from the same family, etc.), developing a framework for improving the performance of committees, and holding workshops to train committee members on their duties. WRA Nigeria also led efforts to develop a unified reporting template for committees that met the needs of government and donors.

During the second phase of the campaign, WRA Nigeria is very focused on helping the ward health development committees to function independently. To do so, they have developed a matrix that the state can use to measure the functionality of committees and then identify where to take action as necessary. Oversight of the committees has become crucial given that they are a signatory to the funds from the Basic Health Care Provision Fund. Such tracking and oversight, however, requires significant amounts of reporting. Each committee is expected to report monthly to the health educator in their local government area. Then 274 reports (one from each ward) ideally trickle up to the state level, where the state health educator must collate them all, and transfer them to the ward health development committee coordinator. This process is cumbersome, and ward health development committees reported frustration about not receiving a response from the state on the contents of their reports.^24^

Not all ward health development committees are equally active, demonstrating the challenges to making them an effective accountability agent. By the end of 2020, only half of the committees in WRA Nigeria’s six target areas were functional,^25^ due primarily to the types of challenges inherent to health facility committees described in the background section.

### Outcomes

Although the purpose of the analysis is to describe the process through which WRA Nigeria sought to build social accountability and collective action around maternal health, there is some evidence of increased government support and citizen engagement that appears related to WRA Nigeria’s activities.

Towards the end of the first phase of the campaign, the governor and deputy governor made specific commitments to involvement in town halls and monitoring of health facilities, and the State Ministry of Health agreed to have citizens involved in monitoring the disbursement of the Basic Health Care Provision Fund. Although responses, rather than responsiveness, these actions had symbolic value, and became concrete items for which citizens and WRA Nigeria advocated. In one case, a citizen journalist complained during a town hall of a dilapidated facility. The Commissioner of Health, who was present at the town hall, asked to see evidence and took down the citizen journalist’s number. Following the town hall, the citizen journalist returned to the facility, took pictures, and then sent them to the Commissioner.

Several anecdotes about responsiveness also emerged. For example, following one town hall, the government supplied necessary equipment to 10 facilities in Chanchaga Local Government Area; the equipment had been procured prior to the town hall, but was sitting unused in the state medical store. In another case, a citizen journalist found no staff person in the waiting room at the hospital. He sent a photo of the empty desk to a friend at the Ministry of Health who then took action.^26^ Following WRA Nigeria’s capacity building workshop for ward health development committees, which was also attended by representatives of the State Ministry of Health, community members reported three health centers without light, leading the Primary Health Care Development Agency Executive Director to have the electric utility fix the problem.^27^

In addition to the well-attended community dialogues and town halls, citizen journalist publications also reflected citizen engagement. Two of the professional citizen journalists published articles perceived by the government as quite critical, some of which are no longer available online. One of these stories was on health budget tracking, and the other covered a variety of issues, including a dilapidated health facility, a nurse who saw 80 patients in a day, and the Ministry of Health not spending funds allocated in the budget [69]. The professional journalists and WRA Nigeria staff reported that over the course of the campaign, the government became more accepting of critical media coverage of health issues.

Challenges also emerged that cut across efforts to build government support, engage citizens, and institutionalize accountability processes. These included the multiplicity of externally funded NGOs working on health issues in the state, who all wanted to meet with the same actors within the Ministry of Health and State Primary Health Care Development Agency. COVID of course complicated efforts, forcing meetings online in a setting with insufficient internet supply, thus increasing data costs for people using their phones to attend virtually. COVID prevented community volunteers from gathering people together and limited the ability of ward health development committees to convene. WRA Nigeria reported that with “online coaching,” however, by June 2020 more than half of committees restarted their monthly meetings.^28^

## Discussion

WRA Nigeria’s campaign has taken a strategic approach to facilitating social accountability by working to both mobilize citizens to demand and hold government accountable, and to increase the state’s willingness to respond. Looking across the two phases of the campaign points to successes and challenges. Successes included convincing the government to accept citizen-led accountability and provide some response to citizens, mobilizing citizens to demand for services and accountability from the state, and increased capacity of the ward health development committees.

Challenges included the constraints that prevented the government from moving towards greater responsiveness in Fox’s framework, which included budgetary factors in particular, but also insufficient prioritization of maternal health by those with the power to effect change. Other challenges included the significant amounts of time WRA Nigeria put towards community mobilization efforts related to community dialogues, town halls, citizen journalists, and community volunteers. To a certain extent WRA Nigeria responded to these challenges by shifting efforts towards institutionalizing the ward health development committees as a lasting accountability mechanism. But community volunteers are needed to monitor the ward health development committees, and the ward health development committees themselves continue to require significant investments of WRA Nigeria staff time, which is occurring only in WRA Nigeria’s six local government areas (out of the total 25 in the state).

Returning to the distinction between government response and responsiveness [31], most of the Niger State government’s actions have fallen under the category of response. The few that are edging towards responsiveness are in progress, and still require significant time inputs from both the state and citizens to become institutionalized. The success of entities such as ward health development committees also depends on many factors largely beyond the state’s control. For example, the Niger State government was happy to have WRA Nigeria train ward health development committees and develop accountability frameworks as doing so helped them make good on their own commitments to the federal government and donors. Moving these responses towards responsiveness will take continued citizen demand, backed up with advocacy support from organizations like WRA Nigeria but also ideally other civil society organizations in Niger State.

This study has several limitations. First, it is not an evaluation of WRA Nigeria’s campaign, so the research design does not permit causal claims about particular elements working or not. Second, the authors’ positionality, as described in the methods section, could raise concerns about an overly positive analysis, but given the study was not an evaluation, we feel that the insider perspective strengthens the analysis. Third, we lack information on any of the downstream, health effects of the campaign. If any emerge, they will take time, and will certainly be due to multiple factors.

## Conclusion

In Niger State, like many places in sub-Saharan Africa, both civil society and the state lack capacity and there is a weak health system. An analysis of WRA Nigeria’s campaign there demonstrates that citizens are willing to participate in community mobilization efforts and demand better health care services. WRA Nigeria’s support has included intensive engagement with the government of Niger State to gain their participation in accountability activities, as well as facilitation of community dialogues, town halls, and the training of citizen journalists and community volunteers. The campaign has convinced the previously unwilling state government to engage with citizens.

For organizations interested in implementing strategic social accountability initiatives, this case study provides several lessons. First, such initiatives require large investments of time and resources by multiple parties. WRA Nigeria staff spent countless hours explaining and promoting the idea of social accountability for health to government and citizen actors alike. Bringing people together to identify and make demands was a significant logistical endeavor, and institutionalizing accountability structures like ward health development committees required engaging with numerous, complex systems. Second, organizations need to be willing and ready to adapt, as demonstrated by WRA Nigeria’s compromise over accountability terminology and their shift away from community dialogues and town halls. Third, WRA Nigeria is active at national, state, and local levels of governance, which helped them frame their campaign in terms that made sense to actors within state ministries but also citizens.

On the whole, the first two phases of the campaign suggest that some groundwork now exists for social accountability for maternal health in Niger State. The question remains about its applicability to social accountability for sexual and reproductive health. From the perspective that social accountability is fundamentally about altering power relations [19, 33, 34], the process has only just begun in Niger State. The experience gained by citizens and the state with social accountability for maternal, newborn, and child health—a relatively safer topic given the sociocultural environment—can only benefit any future steps towards social accountability explicitly oriented towards sexual and reproductive health. Furthermore, given the What Women Want Campaign revealed that women’s primary demands around reproductive and maternal health were WASH and respectful care, promoting social accountability as a means to achieve those goals makes the most sense as a pathway towards improved sexual and reproductive health.

Future social accountability initiatives would be strengthened if complemented by more systematic budget monitoring and the application of open government principles, in line with the Nigerian government’s participation in the Open Government Partnership. Lobbying the governor to share the weekly state budget release, as occurred in Kano State under Governor Kwankwaso [70], would provide unique opportunities for tracking. Technology is also a possible strategy for helping facilitate citizen monitoring, particularly as the Basic Health Care Provision Fund finally begins to transmit resources to facilities. Citizen journalists as well as community volunteers and citizens could thus benefit from apps or other platforms to facilitate such reporting as organizations in other countries have developed [71]. Such technology could increase the number of Niger State citizens able to monitor health facilities, particularly in hard-to-reach parts of the state. Technology is, however, by no means a panacea for accountability failures [72]. At the end of the day, the continued institutionalization of effective accountability structures, such as ward health development committees, as well as any steps that increase the government’s capacity for actual responsiveness to citizen demands, remain central to improving both levels of accountability and citizens’ health in Niger State.

## Data Availability

Data sharing is not applicable to this article as no datasets were generated or analyzed during the current study.

## Abbreviations

NGO: Nongovernmental organization
WASH: Water, sanitation, and hygiene
WRA Nigeria: White Ribbon Alliance Nigeria

## Footnotes

1 Interview #28, NGO. (Details on data collection are described in the methods section below.)

2 Interview #33 – Ministry of Health. The Gates Foundation selected Niger State as a primary health care focal state (along with Kaduna) and signed a memorandum of understanding directly with the state in 2017.

3 June 2020 WRA Nigeria monthly report.

4 Interview #6 – citizen journalist; Interview #8 – Ministry of Health.

5 April 2017 WRA Nigeria monthly report.

6 WRA Nigeria October/November 2020 monthly report.

7 Interview #27 – elected official.

8 Interview #35 – traditional leader.

9 Interview #6 – citizen journalist.

10 Interview #28 – NGO.

11 Interview #41 – WRA Nigeria staff.

12 Interview #35 – traditional leader.

13 Interview #2 – citizen journalist.

14 Interview #7 – traditional leader.

15 Interview #38 – Ministry of Health/donor.

16 Interview #32 – citizen journalist.

17 Interview #42 – citizen journalist.

18 Interview #16 – citizen journalist.

19 Interview #16 – citizen journalist.

20 Interview #21 – citizen journalist.

21 Interview #31 – citizen journalist.

22 Interview #42 – citizen journalist.

23 Interview #45 – WRA Nigeria staff.

24 WRA Nigeria October/November 2020 monthly report.

25 WRA Nigeria November/December 2020 monthly report.

26 Interview #42 – citizen journalist.

27 Interview #23 – Primary Health Care Development Agency; WRA Nigeria November 2017 monthly report.

28 June 2020 WRA Nigeria monthly report.
