# Correction: Building social accountability to improve reproductive, maternal, newborn and child health in Nigeria

**DOI:** 10.1186/s12939-022-01668-7

**Published:** 2022-05-11

**Authors:** Rachel Sullivan Robinson, Tariah Adams

**Affiliations:** 1 School of International Service & Accountability Research Center, American University, 4400 Massachusetts Avenue NW, Washington, DC 20016-8071 USA; 2 White Ribbon Alliance Nigeria, Abuja, Nigeria

**Correction to: Int J Equity Health 21, 46 (2022)**

**https://doi.org/10.1186/s12939-022-01643-2**

Following publication of the original article [1], Fig. 1 and Fig. 2 were shown incorrectly due to a typesetting error. The original article [1] has been corrected.
